# Editorial: Adverse Effects of Cancer Chemotherapy: Anything New to Improve Tolerance and Reduce Sequelae?

**DOI:** 10.3389/fphar.2018.00245

**Published:** 2018-03-22

**Authors:** Kulmira Nurgali, R. Thomas Jagoe, Raquel Abalo

**Affiliations:** ^1^College of Health and Biomedicine, Victoria University, Melbourne, VIC, Australia; ^2^Department of Medicine, Western Health, Faculty of Medicine, Dentistry and Health Sciences, The University of Melbourne, Regenerative Medicine and Stem Cells Program, Australian Institute for Musculoskeletal Science (AIMSS), Melbourne, VIC, Australia; ^3^McGill Cancer Nutrition Rehabilitation Program and the Peter Brojde Lung Cancer Centre, Jewish General Hospital, Montreal, QC, Canada; ^4^Área de Farmacología y Nutrición, Departamento de Ciencias Básicas de la Salud, Universidad Rey Juan Carlos, Alcorcón, Spain; ^5^Unidad Asociada I+D+i del Instituto de Química Médica (IQM), Consejo Superior de Investigaciones Científicas (CSIC), Madrid, Spain; ^6^Unidad Asociada I+D+i del Instituto de Investigación en Ciencias de la Alimentación (CIAL), Consejo Superior de Investigaciones Científicas (CSIC), Madrid, Spain; ^7^Grupo de Excelencia Investigadora URJC, Banco de Santander, Grupo Multidisciplinar de Investigación y Tratamiento del Dolor (i+DOL), Alcorcón, Spain

**Keywords:** chemotherapy, adverse effects, toxicity, cancer treatment, antineoplastic drugs

## Introduction

The side-effects and long-term sequelae of anti-cancer chemotherapy remain a major source of concern for both patients and clinicians despite the improved efficacy and enhanced survival offered by modern treatments. Current drugs or other approaches to counteract chemotherapy-induced adverse effects are often incompletely effective, frequently do not address potential longer-term sequelae or may even induce other side-effects which only add to patient discomfort. New approaches to improve tolerance and reduce sequelae of cancer chemotherapy are urgently needed and the present Research Topic focuses on this issue and highlights several areas of progress.

Nausea and vomiting are amongst the most feared side-effects for patients embarking on cancer chemotherapy. Though current treatments to control acute chemotherapy-induced nausea and vomiting (CINV) are reasonably effective in most patients, delayed CINV is more difficult to manage. The review by Rapoport describes the pathogenesis, incidence and current treatment of delayed CINV, and highlights that this symptom is frequently underestimated and often poorly controlled, even when acute CINV is adequately managed. The release of substance P and its effect on neurokinin-1 (NK-1) receptors is a key step in the development of delayed CINV. Rudd et al. describe pre-clinical studies in animal models (ferrets, house musk shrews) of one NK-1 antagonist, netupitant, as a broad antiemetic (i.e., not only for CINV). In fact, these studies paved the way for the incorporation of this particular drug to the clinic. One of the drawbacks of rodent models in the development of antiemetic drugs is that rodents lack the emetic reflex. However, indirect markers may be used (Andrews and Sanger, 2014) and Yamamoto et al. describe a new potential indirect marker of nausea-like behavior based on monitoring facial expression in the rat. These authors showed the ratio between longitudinal and axial eye dimensions (eye-opening index) decreased after cisplatin administration and this effect was inhibited by conventional antiemetics.

Other gastrointestinal side-effects of cancer chemotherapy are also common and can be both distressing and potentially fatal for patients. In their review, Cinausero et al. describe the pathobiology and treatment of cancer treatment-related mucosal injury. Both oral and gastrointestinal mucositis may cause local ulceration and pain, which in turn may lead to anorexia, malabsorption, weight loss, anemia, fatigue and increased risk of sepsis. It is important to note that despite much prior research on oral mucositis, safe and effective preventive measures and treatments are still lacking. This likely reflects the complexity of the pathobiology of gastrointestinal mucositis, and highlights the fact that mucosal injury probably contributes to other chemotherapy-induced gastrointestinal disorders. McQuade et al. describe the pathophysiology, and current and emerging treatments for chemotherapy-induced constipation (CIC) and diarrhea (CID), both of which are common and may require dose reductions, delays or even cessation of treatment. CID is potentially fatal due to dehydration and electrolyte imbalance and current therapeutic approaches include re-hydration, loperamide, and octreotide. However, pre-clinical and clinical studies, of new treatments for CID are described. These include inhibitors of calcium-activated chloride channels, β-glucuronidase inhibitors, antibiotics, probiotics, and cannabinoid agonists. The authors also emphasize that CIC is more frequent and severe than commonly recognized. Over-use of anti-diarrhoeal treatments for CID is one frequent cause of CIC, but other mechanisms of CIC are not well understood. Current treatments often include laxatives and certain prokinetic agents. However, agonists targeting intestinal guanylate cyclase C or chloride channels show promise as potential targets for future studies for CIC. Interestingly, using radiographic methods in rats, Vera et al. showed that a CB1 receptor cannabinoid antagonist prevented the effect of acute vincristine on gastrointestinal motility, particularly in the small intestine. Thus, inactivation of the cannabinoid system might be useful to counteract CIC, whilst cannabinoid receptor activation might be used to counteract CID (Abalo et al., 2017; McQuade et al.).

With the expanded use of anti-cancer treatments in different groups of patients, the profile of toxicities associated with well-established agents, such as platinum-based chemotherapies, continues to broaden. One example, namely the occurrence of hypersensitivity reactions to carboplatin in children being treated for solid tumors such as low-grade glioma, is reviewed by Ruggiero et al. Studies show that such hypersensitivity reactions occur in up to 47% of children treated with this agent. Younger children, girls and those with other allergies are at higher risk and the incidence rises with increased number of infusions rather than simply drug dosage. Another platinum-based chemotherapeutic, cisplatin, may increase the risk of cardiovascular disease in cancer survivors. Herradón et al. explored the possible mechanisms for this, using 5 weekly intraperitoneal injections of cisplatin, in male Wistar rats. In their model, there was evidence of vascular endothelial changes at lower doses and impacts on cardiac function at the highest dose. In contrast to cardiovascular toxicity, cisplatin-induced nephrotoxicity is well recognized and Malik et al. report encouraging results for a potential protective effect of a botanical, *Emblica officinalis* (Indian gooseberry). Premedication with *E. officinalis* protected male Wistar rats from nephrotoxicity with reduction in the inflammation and oxidative damage induced by a single intraperitoneal injection of cisplatin. Evidence of chronic subclinical skeletal muscle toxicity from chemotherapy is accumulating and this has important implications for longer-term health status for large numbers of cancer survivors. Unfortunately, there is still a lack of detailed mechanistic studies investigating the potential impact of anti-cancer agents on skeletal muscle but the manuscript by Sorensen et al. is the first to describe the direct effects of repeated oxaliplatin dosing on skeletal muscle, including aspects of skeletal muscle mitochondrial function. In addition, they show that the small molecule, BGP-15, protects against oxaliplatin-induced muscle wasting, muscle collagen deposition and changes in muscle mitochondrial function in their model which uses male BALB/c mice receiving six intraperitoneal injections over 12 days.

Central and peripheral neurotoxicity caused by anti-cancer drugs can last many years after the end of treatment and can dramatically reduce functional capacity and quality of life in cancer survivors. A review of clinical studies on biological markers associated with cognitive impairments in cancer patients during and after chemotherapy by Castel et al. is included in this Research Topic. In it the authors identified studies showing changes in a number of circulating factors and cerebrospinal fluid constituents which were associated with chemotherapy-induced persistent cognitive dysfunctions. These factors along with genetic polymorphisms might be used as predictive markers to identify patients predisposed to cognitive deficits caused by chemotherapy.

Chemotherapy-induced peripheral neuropathy (CIPN) is caused by many anti-cancer drugs including platinum-based agents, vinca alkaloids, taxanes, and proteasome and angiogenesis inhibitors. Long-term CIPN is associated with high morbidity including depression, ataxia, insomnia. Kerckhove et al. provide a comprehensive review of pathophysiological mechanisms, symptoms and risk factors of long-term CIPN induced by specific types of chemotherapeutic drugs. However, prevention and treatment strategies for long-term CIPN are not well-developed and are urgently needed. Thus, it is gratifying to include two original studies on this issue, in this Research Topic. Sundar et al. present a pilot clinical trial assessing limb hypothermia to prevent CIPN induced by paclitaxel in breast cancer patients. Using nerve conduction recording, the activities of several sensory and motor nerves were evaluated before, during and after chemotherapy. The results of this study suggest that continuous-flow limb hypothermia can preserve specific parameters of nerve conduction and significantly benefit some patients undergoing paclitaxel chemotherapy. These results are supported by another pilot study providing evidence that limb hypothermia has a potential to alleviate paclitaxel-induced symptoms of peripheral neuropathy in breast cancer patients (Younus et al., 2016). Kim et al. demonstrated that systemic administration of a reactive oxygen species scavenger, tempol, which has previously been shown to be of benefit in a rat model of cancer-induced bone pain (Zhou et al., 2018), also ameliorated and prevented neuropathic pain induced by paclitaxel in rats. As mentioned by McQuade et al. there is evidence that chemotherapy-induced enteric neuropathy may contribute to the occurrence of permanent gastrointestinal dysfunction in cancer survivors. Thus, further studies of neuroprotective agents to combat this type of neurotoxicity may also be warranted.

One of the most startling and exciting changes in cancer treatment over recent years has been the emergence of therapies aimed at enhancing the patient's own immune response to their tumor. The immune checkpoint inhibitors are now well-established in the treatment of malignant melanoma and rapidly expanding their role in the treatment of many other tumors. However, whilst these agents do not induce severe acute nausea, vomiting or marrow suppression associated with many traditional cytotoxic agents, it is becoming clearer that they can lead to a whole range of other immune–related side-effects in many different organs. Such side-effects are sometimes challenging to identify but can be life-threatening. Kumar et al.; Kumar et al. provide a detailed and timely review of current knowledge about these immune-related adverse events and a framework for clinical management. Other therapeutic approaches, using cell-based therapies to enhance host immune response to tumors, are also being actively pursued. Mosińska et al. describe the potential for a combination of host dendritic cells and cytokine-induced killer cells that are primed to target and kill cells expressing tumor antigens. As the authors explain, the specificity offered by killing only cells expressing tumor antigens is potentially a very powerful way to avoid side-effects from other less targeted cytotoxic treatments. However, for now it is too early to say whether the promising results from early trials will be fulfilled.

Finally, the combination of natural bioactive compounds with traditional chemotherapeutic drugs can potentiate anti-cancer efficacy and reduce side-effects of chemotherapy. In some cases, addition of bioactive compounds may overcome the chemo- or radio-resistance of cancer cells. These synergistic effects of nutraceutical compounds such as flavonoids, stilbenes, terpenes, curcumin, and others have been discussed in a review article by Redondo-Blanco et al. presented in this Research Topic. The authors reviewed current knowledge on mechanisms of action of these compounds based on studies in colorectal cancer cells, animal models and clinical trials. However, the use of non-approved combinations of drugs and unproven remedies may lead to severe side-effects and life-threatening toxicities. Uhl et al. present a case report on fatal toxicity induced by a combination of dichloroacetate and artemisinin derivative, artesunate. Both drugs exert anti-cancer activity *in vitro* and *in vivo*, and were trialed in a small number of cancer patients; however, a combination of these drugs provoked severe liver and bone marrow toxicity in the patient. The authors discuss the literature on the side-effects of these drugs.

In conclusion, the present Research Topic has already generated a lot of interest with high numbers of views and citations, but there are still many aspects of this topic area that deserve further attention. These include the impact of cancer chemotherapy on sensory functioning such as hearing, approaches to maintain fertility during and after treatment and the broad long-term impact of systemic anti-cancer treatment on health and aging in cancer survivors (Cupit-Link et al., 2017). We look forward to more new studies to answer these and many other related questions and we anticipate that this will continue to be a dynamic and expanding area of research. Finally, we hope that by identifying and minimizing or preventing both short and longer-term toxicity from cancer chemotherapy, the treatments themselves will be better tolerated and more effective, and the health and wellness of cancer survivors will be enhanced.

## Author contributions

All authors listed have made a substantial, direct and intellectual contribution to the work, and approved it for publication.

### Conflict of interest statement

The authors declare that the research was conducted in the absence of any commercial or financial relationships that could be construed as a potential conflict of interest.
